# A Novel Mutation of *GFAP* Causing Adult-Onset Alexander Disease

**DOI:** 10.3389/fneur.2019.01124

**Published:** 2019-11-06

**Authors:** Andrea Ciammola, Davide Sangalli, Jenny Sassone, Barbara Poletti, Laura Carelli, Paolo Banfi, Gabriele Pappacoda, Isabella Ceccherini, Alice Grossi, Luca Maderna, Monica Pingue, Floriano Girotti, Vincenzo Silani

**Affiliations:** ^1^Department of Neurology and Laboratory of Neuroscience, Istituto di Ricovero e Cura a Carattere Scientifico (IRCCS), Istituto Auxologico Italiano, Milan, Italy; ^2^Istituto di Ricovero e Cura a Carattere Scientifico (IRCCS), San Raffaele Scientific Institute, Milan, Italy; ^3^Vita-Salute San Raffaele University, Milan, Italy; ^4^Istituto di Ricovero e Cura a Carattere Scientifico (IRCCS), Fondazione Don Carlo Gnocchi, Milan, Italy; ^5^Istituto di Ricovero e Cura a Carattere Scientifico (IRCCS), Istituto Giannina Gaslini, Genoa, Italy; ^6^Department of Pathophysiology and Transplantation, Dino Ferrari Center, University of Milan, Milan, Italy

**Keywords:** Alexander disease, GFAP-glial fibrillary acidic protein, leukodystrophy, gene mutation, adult onset

## Abstract

Alexander disease (AxD) is a rare, autosomal dominant neurological disorder. Three clinical subtypes are distinguished based on age at onset: infantile (0–2 years), juvenile (2–13 years), and adult (>13 years). The three forms differ in symptoms and prognosis. Rapid neurological decline with a fatal course characterizes the early-onset forms, while symptoms are milder and survival is longer in the adult forms. Currently, the sole known cause of AxD is mutations in the *GFAP* gene, which encodes a type III intermediate filament protein that is predominantly expressed in astrocytes. A wide spectrum of *GFAP* mutations comprising point mutations, small insertions, and deletions is associated with the disease. The genotype-phenotype correlation remains unclear. The considerable heterogeneity in severity of disease among individuals carrying identical mutations suggests that other genetic or environmental factors probably modify age at onset or progression of AxD. Describing new cases is therefore important for establishing reliable genotype-phenotype correlations and revealing environmental factors able to modify age at onset or progression of AxD. We report the case of a 54-year-old Caucasian woman, previously diagnosed with ovarian cancer and treated with surgery and chemotherapy, who developed dysarthria, ataxia, and spastic tetraparesis involving mainly the left side. Cerebral and spinal magnetic resonance imaging (MRI) revealed a peculiar tadpole-like atrophy of the brainstem. Genetic analysis of the *GFAP* gene detected a heterozygous mutation in exon 1 (c.219G>C), resulting in an amino acid exchange from methionine to isoleucine at codon 73 (p.M73I). The expression of this mutant *in vitro* affected the formation of the intermediate filament network. Thus, we have identified a new *GFAP* mutation in a patient with an adult form of AxD.

## Background

Alexander disease (AxD) is an autosomal dominant neurological disorder (OMIM **#**203450) caused by mutations in the glial fibrillary acidic protein (*GFAP*) gene located on chromosome 17q21.31. AxD is a leukodystrophy characterized by the progressive accumulation in astrocytes of GFAP aggregates, called Rosenthal fibers, which are spread over the subpial, perivascular, and subependymal regions of the cortex and white matter. These inclusions impair cytoskeleton formation and astrocyte survival and functioning, ultimately resulting in demyelination (1).

AxD is extremely rare. According to the only population-based study completed to date, the estimated 5-year prevalence is 1 in 2.7 million (1, 2). Three clinical subtypes are distinguished based on age at onset: infantile (0–2 years), juvenile (2–13 years), and adult (>13 years). The early-onset forms are characterized by progressive neurological decline, with developmental delay, pyramidal sign, seizure, ataxia, and a fatal course; the median survival is 14 years (3). The adult form is characterized by normal cognition, swallowing difficulties, dysarthria, nystagmus, palatal myoclonus, pyramidal dysfunction, and cerebellar ataxia; the median survival is 25 years. The symptoms reflect the typical, marked atrophy and hyperintensity signal in the lower brainstem, the upper cervical cord, the dentate nucleus, and the middle cerebral peduncles frequently observed on magnetic resonance imaging (MRI) in patients with adult-onset AxD (4).

A wide spectrum mutation in the *GFAP* gene is associated with the disease (https://www2.waisman.wisc.edu/alexander-disease/mutation-table.pdf) and the genotype-phenotype correlation is still unclear. There are mutations that cause both infantile and juvenile-onset forms and other mutations that cause all three forms (1, 5, 6). Single *GFAP* mutations cause heterogeneous neurological symptoms that can vary in severity among members of the same family (6). The progression rate also is variable, with a case of self-remitting AxD described in the literature (7). This heterogeneity suggests that still unknown genetic or environmental factors probably modify age at onset or progression of disease. Describing new cases is therefore important. Here we describe the onset and progression of neurological symptoms in an adult patient bearing a new mutation of the *GFAP* gene. We also demonstrate *in vitro* that this novel mutation affects the formation of the intermediate filament (IF) network.

## Materials and Methods

### Clinical Assessment and Brain Imaging

The study was approved by the Ethics Committee of the IRCCS Istituto Auxologico Italiano. Written, informed consent for this study and for publication of this case report was obtained from the patient. The patient underwent 3T brain MRI (Siemens Scan). Lung function was assessed using a non-contact non-invasive system (Thora-3DI™, PneumaCare Limited) with the patient in supine position at rest.

### Genetic Analysis

DNA was extracted from peripheral blood. The nine exons, and corresponding exon–intron boundaries of the *GFAP* gene, isoform 1 (RefSeq NM_002055.5) were amplified and sequenced as described previously (8). The polymerase chain reaction (PCR) amplification reactions were performed on ABI 9700 and/or ABI 2700 thermal cyclers. The following primers were used for PCR amplification: exon1FOR ctccttcataaagccctcgc, exon1REV gatagtgccccatcaagagg, exon2-3FOR aggcaggtattcaagtgtcc, exon2-3REV atttggtgtctctacctgcc, exon4FOR caagagagcattcgaactcc, exon4REV aggatattctcccagcttcc, exon5-6FOR gtgttgtgctaggtgctgagg, exon5-6REV gtgactgcctgctatgtgtgagg, exon7FOR gctaggagatggagttagac, exon7REV aagtaccctggtatgataggc, exon8FOR ctgctcggttgcataggttc, exon8REV gctgggaaccttctatgtgc, exon9FOR tcctaactgttgcactgtgc, exon9REV gagcaactatcctgcttctg. Temperature profiles for all PCR amplifications were: initial 5 min denaturation at 95°C, followed by 30 cycles at 96°C for 30 s, 55°C for 20 s, 72°C for 45 s, and a final 5 min extension at 72°C. All reaction conditions were optimized to obtain substantial uniformity of annealing temperature (55°C) and Mg^2+^ concentration (1.5 mM). Subsequently, 10 μl of the PCR products were separated and purified from single-stranded DNA chains and non-linked oligonucleotides by enzymatic digestion with exonuclease I and shrimp alkaline phosphatase combined in an Exo-SapIT kit (USB-Amersham). Purified PCR products were then directly sequenced using the BigDye™ Terminator v3.1 Cycle Sequencing Kit (ThermoFisher Scientific) and run on an ABI PRISM 3100 Genetic Analyzer (ThermoFisher Scientific).

### Cellular Assay

Plasmid encoding human *GFAP* was purchased from OriGene Technologies (SC118873). The point mutations M73I and R239C were introduced (QuikChange XL Site-Directed Mutagenesis Kit, Agilent) and verified by Sanger sequencing. HeLa cells were grown in Dulbecco's modified Eagle's medium, 10% fetal bovine serum, 1% Pen-Strep, 5% CO_2_ at 37°C, and transfected using lipofectamine 2000; 48 h after transfection the cells were immunostained with mAb #3670 Cell signaling GFAP (1: 300). Fluorescence images were acquired using a confocal microscope (Leica TCS). Image acquisition and morphometric quantification were performed by investigators blinded to the experimental condition.

## Case Presentation

### Clinical Reports

A Caucasian woman, born in 1960, was diagnosed with ovarian cancer (pT3cN1G3) in 2008 and treated with surgery, carboplatin, and paclitaxel. During the second and third chemotherapy cycles (seven cycles in total), her relatives noted onset of an imperfect utterance of words and an unstable and wide-base gait. Since the patient initially thought her symptoms were adverse effects of the chemotherapy, she did not ask for neurological evaluation. In 2009 her neurological symptoms suddenly worsened. Neurological assessment carried out at the emergency department disclosed asymmetric spasticity, with mild left-limb weakness, dysarthria, and ataxia. Computed tomography (CT) scan of the brain revealed no acute ischemic damage or hemorrhage. The patient refused further clinical investigations. A few months later, she developed diaphragmatic metastasis and underwent surgery followed by hyperthermic intraperitoneal chemotherapy with cisplatin and mitomycin C. Since her neurological symptoms remained generally unchanged, neurological investigations were delayed further.

**Figure 1:**
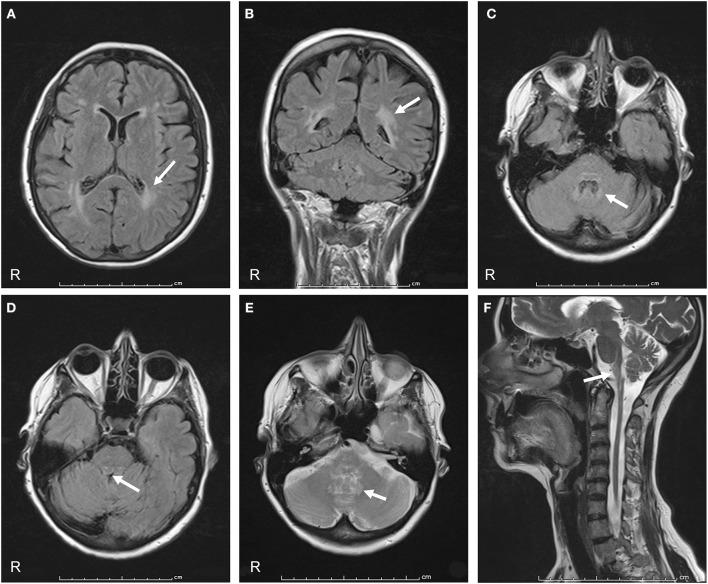
Axial and coronal FLAIR MRI show signal hyperintensity prevalent in the cerebral posterior periventricular regions (arrows in **A,B**) and in the hilum of the dentate nuclei (arrow in **C**). Midbrain and pons show peripheral rim of hyperintensity (arrow in **D**). Axial T2-weighted sections show increased signal intensity in the hilum of the dentate nuclei (arrow in **E**). A sagittal T2-weighted image shows atrophy and signal change in the medulla and spinal cord, “tadpole sign” (arrow in **F**).

On admission to our hospital in 2014, neurological examination revealed slurred speech, mild dysphagia, spastic tetraparesis involving mainly the left side, increased muscle stretch reflexes and Babinski sign, bilateral horizontal nystagmus, dysmetria, ataxia, and moderate dysdiadochokinesia. Cognitive testing was normal. Review of the family history disclosed that her father had a gait disturbance, diagnosed as a possible post-polio syndrome, and her 56-year-old sister had a gait disturbance, as well. No further family information was available. Hematologic screening and cerebrospinal fluid (CSF) examination were normal. No paraneoplastic antibodies (anti-Yo, -Hu -Ri) were detected in serum or CSF. Cerebral and spinal MRI showed marked atrophy from the medulla oblongata to the upper cervical spinal cord with dorsal enhancement. The pons was slightly atrophic, with a peculiar tadpole-like atrophy of the brainstem. Periventricular hyperintensity was observed on fluid attenuation inversion recovery (FLAIR) MRI (Figure 1). Overall, the clinical and radiological features were suggestive of AxD. Genetic analysis disclosed a novel missense mutation in the *GFAP* gene (see Genetic Findings).

The neurological examination performed in 2018 showed the onset of weakness in the sternocleidomastoid, trapezius, and proximal upper limb muscles. Because of severe worsening of lower limb spasticity, the patient used a wheelchair. The neurological examination also showed the onset of a palatine tremor (at ≈2 Hz) and square waves jerks. A formal neuropsychological evaluation disclosed selective impairment of executive and attentional abilities, together with mild involvement of verbal memory, while the other cognitive functions and global cognition were preserved (Mini Mental State Examination raw score 28/30). Impulsivity was noted during cognitive testing. A formal assessment of behavior (Frontal Behavioral Inventory, FBI) revealed the presence of prevailing negative symptoms (apathy, aspontaneity, inflexibility, and personal neglect) accompanied by irritability (FBI total score 15/72, Negative Behaviors total score 9/36; Disinhibition total score 6/36). Some behavioral features were probably related to the presence of moderate depressive symptoms, as confirmed by the Beck Depression Inventory (BDI total score 27/63). No neurological therapy was administered.

Starting in 2016, the patient developed progressive cough and bronchial asthma in relation to a history of smoking. In January 2019 she was admitted to a respiratory day hospital for acute bronchitis with respiratory failure (arterial blood gases pH 7.36, pCO_2_ 57.0 mm Hg, pO_2_ 54.5 mm Hg, B.E. 11 mmol/L, HCO_3_ 38.6 mmol/L, SO_2_ 84%). Chest X-ray showed no major alterations. The respiratory pattern during bronchial exacerbation was recorded with the patient in supine position at rest (PneumaCare Thora-3DI™). Paradoxical respiration is shown in Supplementary Figure 1. Non-invasive ventilation led to an improvement in respiratory symptoms.

### Genetic Findings

**Figure 2:**
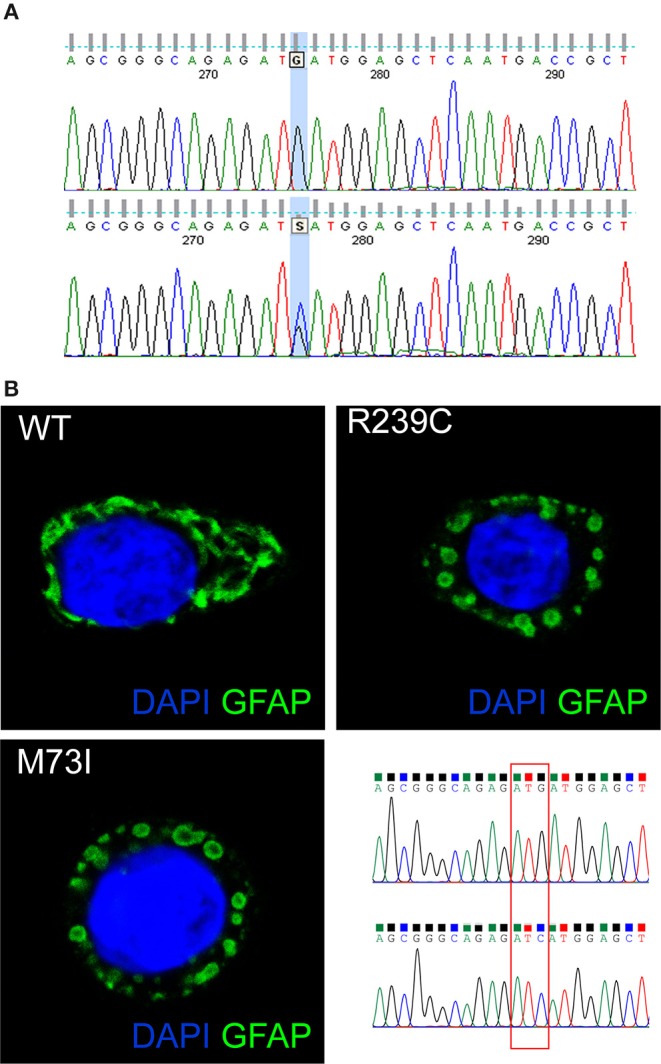
**(A)** The *GFAP* exon 1 sequence, obtained from a control (top) and our patient (bottom). The bottom picture shows heterozygosity for the missense c.219G>C nucleotide change, corresponding to the p.M73I mutation. **(B)** Representative images showing HeLa cells transiently transfected with plasmids encoding human wild-type GFAP or mutant GFAP M73I and R239C and labeled with GFAP antibody. The image shows wild-type GFAP assembled in filament networks, whereas mutant M73I and R239C formed dot-like aggregates. On the right, a detail is given of the Sanger sequences of the plasmids encoding wild-type GFAP and GFAP M73I; codon 73 was mutated from ATG (Met) to ATC (Ile).

Genetic analysis of the *GFAP* gene detected a heterozygous mutation in exon 1 (c.219G>C), resulting in an amino acid exchange at codon 73 from methionine to isoleucine (p.M73I) (Figure 2A). Consistent with highly conserved Met73 (GERP^++^ = 2.26), the present substitution to an isoleucine residue was not found in 125,748 GnomAD exomes and 15,708 GnomAD genomes (https://gnomad.broadinstitute.org/). Three different missense mutations at the same residue of the GFAP protein have been previously reported: p.M73R, p.M73K, and p.M73T. These missense variants are all classified as “likely pathogenic” and, indeed, found only in association with AxD (5, 8, 9) but not in 125,748 GnomAD exomes and 15,708 GnomAD genomes. Coherent with the role the present *GFAP* mutation can play, several *in silico* analyses have predicted the p.M73I substitution to be pathogenic (https://varsome.com/). Since parental screening could not be performed, an either “*de novo*” or inherited nature of the p.M73I mutation remains to be defined.

### *In vitro* Model

GFAP is a key IF protein responsible for the assembly of the cytoskeleton in glia cells. Previous papers showed that AxD-associated mutations perturb IF assembly (1). To test whether this new mutation p.M73I affects IF network formation, we transfected HeLa cells with plasmids that encode human wild-type or mutated GFAP, including the p.M73I, and the previously described AxD mutation p.R239C (https://www2.waisman.wisc.edu/alexander-disease/mutation-table.pdf). Wild-type GFAP assembled into bundled filaments that extended throughout the cytoplasm; the proportion of cells showing GFAP aggregates from the transfected wild-type-GFAP was 0.00%. Both M73I-GFAP and R239C-GFAP failed to assemble into filaments; instead, they formed clusters of cytoplasmic aggregates. The proportion of cells transfected with M73I-GFAP and R239C-GFAP showing GFAP aggregates was 100% (*n* = 170 HeLa cells transfected with wild-type-GFAP, *n* = 148 cells transfected with M73I-GFAP, *n* = 172 cells transfected with R239C-GFAP, Chi-square 490.0, df 2, *p* < 0.0001, Figure 2B).

## Discussion

We have identified a new *GFAP* mutation in a patient with an adult form of AxD. This mutation causes the substitution of the amino acid methionine at codon 73 with an isoleucine (p.M73I). Three different missense mutations at the same residue of the GFAP protein have been reported: p.M73R, p.M73K, and p.M73T. p.M73R was found in a juvenile case of AxD: a boy with disease onset at 9 years of age, affected by dysphagia, frequent episodes of vomiting and choking, dysarthria, slurred speech, and strabismus (9). p.M73K was found in an infantile type of AxD: a male infant with severe symptoms and onset at 7 months of age (8). p.M73T was described in three severe cases of infantile AxD: a male with onset at 3 months of age that presented with seizures and died at 2 years of age (5); a neonate (10) and, in cis with another missense *GFAP* variant, p.V87F, in another case of infantile AxD (11).

We wondered why the p.M73I mutation is associated with a less severe form and adulthood onset. A hydrophobic side chain characterizes methionine. The amino acid changes from Met73 to either Arg or Lys introduce a positive charge, whereas p.M73T introduces a polar amino acid. These charge-altering substitutions may alter the oligomerization or solubility of the GFAP protein. Conversely, because it bears a hydrophobic side chain, isoleucine resembles the methionine structure, leading to few changes in the chemico-physical properties of codon position 73. We speculate that p.M73I leads to an adult form of AxD because its effect on GFAP protein structure is milder than that of mutations p.M73R/K/T.

The patient in the present case had adult onset with typical AxD symptoms, such as dysarthria and ataxia. The early symptoms started during chemotherapy, but a correlation between the chemotherapy and the onset of neurological symptoms is tenuous. A case report described clinical worsening in an infantile form of AxD following cisplatin and vincristine administered in the erroneous suspicion of an astrocytoma (12). Since other studies suggested that environmental factors may cause AxD onset or modify its course (1), we may speculate that the proinflammatory state induced by cancer, in addition to the cytotoxic effects of cancer therapy, may have triggered the disease onset.

The appearance of palatal tremor and square-wave jerks in our patient reflects the progressive damage to the brainstem and cervical cord, a feature typical of AxD. We also noted a potential effect of the palatal tremor on chest expansion during inspiration (Supplementary Figure 1). Whether this effect significantly interfered with breathing in our patient is uncertain, given her history of bronchial asthma. The finding of respiratory involvement in our patient is shared by the observation of previous case reports that sleep apnoea and respiratory dysfunction may appear during the progression of adult onset AxD (13, 14). The cause of respiratory involvement in AxD remains poorly understood: previous studies suggested that epiglottic dysfunction causes obstructive sleep apnoea (14) and that palatal and laryngeal tremor can interrupt laryngeal airflow (13). A larger study investigating respiratory dysfunctions in AxD is needed to clarify these aspects.

MR imaging criteria of AxD in a study of juvenile AxD include extensive cerebral white matter changes with frontal predominance, periventricular rim with high signal on T1-weighted images and low signal on T2-weighted images, abnormalities of basal ganglia and thalami, brain stem abnormalities, and contrast enhancement of particular gray and white matter structures (15). We observed no frontal white matter abnormalities and periventricular involvement was scarce in our patient. These findings agree with a previous description of late-onset AxD in which atypical MRI features were sparse involvement of the periventricular white matter, predominance of posterior fossa white abnormalities, brainstem atrophy, cerebellar atrophy, and spinal cord atrophy (3). In their study of a cohort of 13 patients with late-onset AxD, Graff-Radford et al. found white matter involvement in <50%; the few with severe or moderate white matter involvement had a younger age at onset than our patient (4). Hence, the MRI alterations seen in our patient were milder than those previously described and may reflect the later onset and slow progression of the disease.

In conclusion, we describe the onset and progression of neurological symptoms in an adult patient bearing a new missense mutation of the *GFAP* gene. We also demonstrate *in vitro* that this novel mutation affects the formation of the intermediate filament network.

## Ethics Statement

The studies involving human participants were reviewed and approved by ethics committee of Istituto Auxologico Italiano. The patients/participants provided their written informed consent to participate in this study.

## Author Contributions

AC and JS contributed to the conception and design of the study. AC, JS, IC, DS, FG, BP, LC, PB, GP, AG, LM, and MP contributed to the acquisition of data, or analysis and interpretation of data. AC, JS, IC, and PB contributed to the drafting the article. AC, IC, FG, and VS contributed to the revision for important intellectual content. All authors have approved the final article.

### Conflict of Interest

The authors declare that the research was conducted in the absence of any commercial or financial relationships that could be construed as a potential conflict of interest.
