# Supplementary figures and images for: A Novel Mutation of GFAP Causing Adult-Onset Alexander Disease
Source: Front Neurol. 2019 Nov 6;10:1124. doi: 10.3389/fneur.2019.01124 (PMC6851058; doi:10.3389/fneur.2019.01124)

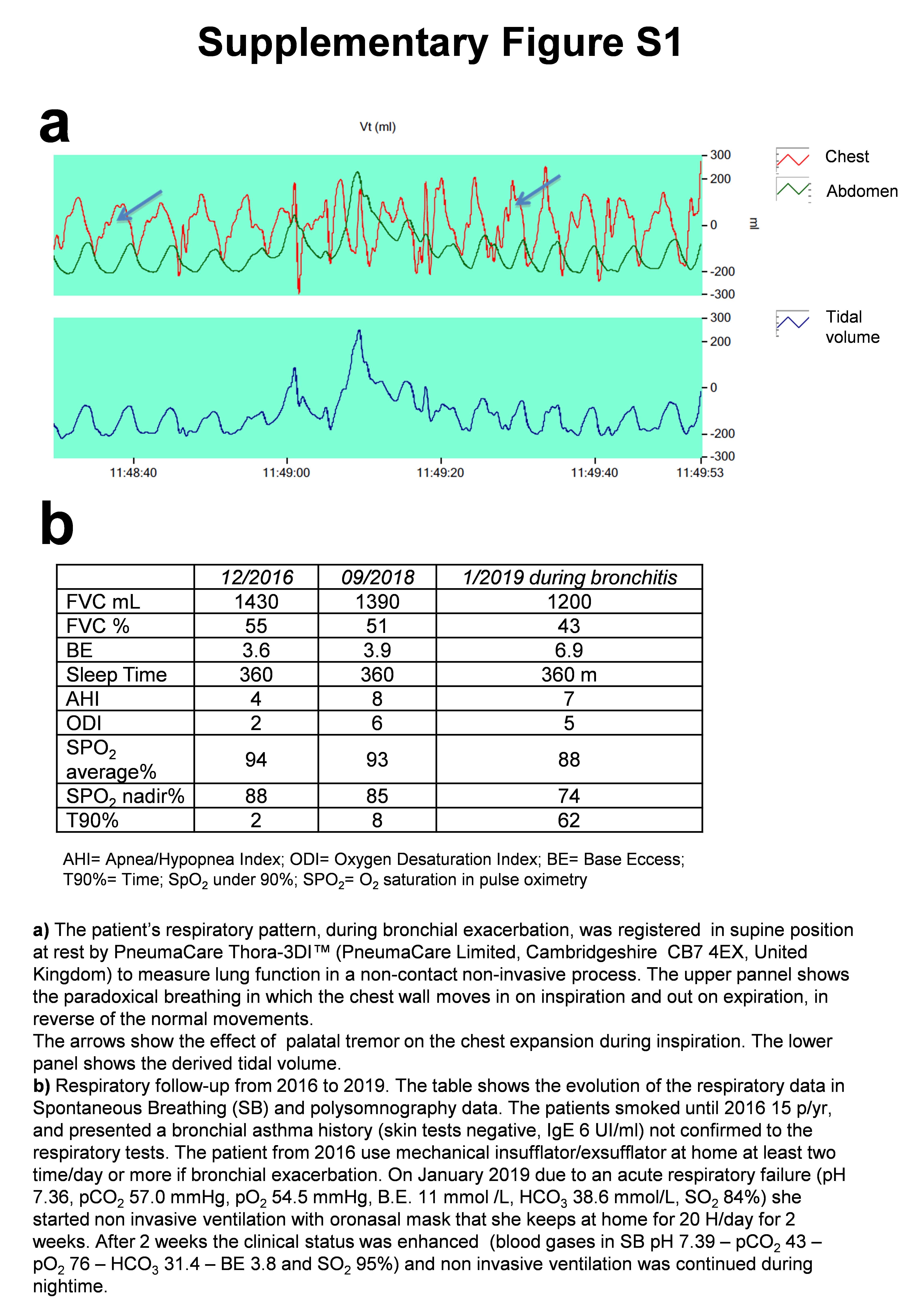

Supplement: Supplementary file 1 [file Image_1.TIF]
